# Effects of Sea-Buckthorn Flavonoids on Growth Performance, Serum Inflammation, Intestinal Barrier and Microbiota in LPS-Challenged Broilers

**DOI:** 10.3390/ani14142073

**Published:** 2024-07-15

**Authors:** Kexin Zhi, Fanwen Gong, Lele Chen, Zezheng Li, Xiang Li, Huadi Mei, Chenxing Fu, Yurong Zhao, Zhuying Liu, Jianhua He

**Affiliations:** 1 College of Animal Science and Technology, Hunan Agricultural University, Changsha 410128, China; 13276901791@163.com (K.Z.); 13667920590@163.com (F.G.); chen1830909@163.com (L.C.); l15093587113@163.com (Z.L.); lxffy0123@163.com (X.L.); m15973699341@163.com (H.M.); chenxing1110@outlook.com (C.F.); zyr@hunau.edu.cn (Y.Z.); 2 College of Animal Science and Technology, Hunan Biological and Electromechanical Polytechnic, Changsha 410128, China

**Keywords:** sea-buckthorn flavone, lipopolysaccharide, inflammation, intestinal health, broilers

## Abstract

**Simple Summary:**

High-density intensive farming easily induces immune stress in broilers, leading to significant impairment of growth performance and intestinal health. Sea-buckthorn flavone (SF), extracted from the fruits, leaves, and branches of sea buckthorn, exhibits anti-oxidative, anti-inflammatory, anti-obesity, liver protective, and lipid metabolism-regulating properties. This study examined the effects of SF on growth performance, serum inflammation, the intestinal barrier, and the microbiota in lipopolysaccharide (LPS)-challenged broilers. Our findings demonstrate that SF alleviates LPS-induced inflammatory responses in broilers while improving intestinal health and enhancing growth performance. These findings serve as a valuable reference for poultry production.

**Abstract:**

The experiment investigated the effects of sea-buckthorn flavonoids (SF) on lipopolysaccharide (LPS)-challenged broilers. A total of 288 one-day-old male broilers were randomly assigned to 4 groups, with 6 replicates of 12 broilers each. The experiment lasted for 20 days. The diet included two levels of SF (0 or 1000 mg/kg) and broilers intraperitoneally injected with 500 μg/kg LPS on 16, 18, and 20 days, or an equal amount of saline. LPS challenge decreased final body weight, average daily gain, and average daily feed intake, increased feed-to-gain ratio, and elevated serum IL-1β, IL-2, TNF-α, D-LA, and endotoxin levels. Moreover, it resulted in a reduction in the IL-10 level. LPS impaired the intestinal morphology of the duodenum, jejunum, and ileum, down-regulated the mRNA relative expression of Occludin, ZO-1, and MUC-2 in the jejunum mucosa, up-regulated the mRNA relative expression of TLR4, MyD88, NF-κB, and IL-1β, and increased the relative abundance of *Erysipelatoclostridium* in broilers (*p* < 0.05). However, SF supplementation mitigated the decrease in growth performance, reduced serum IL-1β, IL-2, and D-LA levels, increased IL-10 levels, alleviated intestinal morphological damage, up-regulated mRNA expression of Occludin and ZO-1, down-regulated the mRNA expression of TLR4, NF-κB, and IL-lβ in jejunum mucosal (*p* < 0.05), and SF supplementation presented a tendency to decrease the relative abundance of proteobacteria (0.05 < *p* < 0.1). Collectively, incorporating SF can enhance the growth performance, alleviate serum inflammation, and improve the intestinal health of broilers, effectively mitigating the damage triggered by LPS-challenges.

## 1. Introduction

Under modern intensive breeding conditions, high breeding density, pathogenic microorganisms, temperature, and humidity factors can induce immune stress in broilers, which results in damage to growth performance and the intestinal barrier in poultry [1,2]. LPS is a common endotoxin and is considered to be one of the most potent immune activators [3]. Previous studies demonstrated that intrabitoneal injection of LPS in broilers could seriously compromise the integrity of the intestinal mucosal barrier, elevate inflammatory factors, alter intestinal flora, and consequently impact growth performance [4,5]. LPS has been shown to activate the NF-κB signaling pathway by binding to the TLR4 receptor on the cell surface to generate a number of pro-inflammatory cytokines [6]. Notably, natural plant extracts contain a variety of nutrients, which can enhance the growth performance and feed utilization of livestock and poultry [7]. Therefore, it is crucial to develop a natural and botanic extract as a feed additive to ameliorate the immune stress of poultry and maintain intestinal health.

Sea-buckthorn (*Hippophae rhamnoides* L.) is a versatile medicinal and edible plant known for its strong adaptability and high yield [8]. It has been reported to possess typical physiological functions, including anti-inflammatory properties [9], antioxidant activity [10], immunomodulatory effects [11], and lipid-lowering capabilities [12]. Studies have indicated the beneficial impact of sea-buckthorn leaves or extracts on growth performance, feed efficiency, and the development of immune organs in Turkey poults, with long-term feeding being deemed safe and reliable [13]. Sea-buckthorn flavonoids (SF) are the major active components extracted from the fruits, roots, and leaves of sea-buckthorn. They may serve as natural feed additives, enhancing growth performance, rumen fermentation, and serum antioxidant capacity in lambs [14]. Furthermore, evidence suggests that key components of sea-buckthorn flavonoids such as quercetin, isorhamnetin, and kaempferol could alleviate inflammation challenged by LPS in mice [15]. Studies have shown that these compounds fight inflammation through different mechanisms [16,17,18]. It is currently attracting attention from researchers due to its extensive physiological functions. However, there is limited data available regarding the regulatory effects of SF on inflammatory responses, the intestinal barrier, and the microbiota in broilers under LPS-challenged immune stress. The present investigation aims to explore the effects of SF on the performance, serum inflammation, intestinal barrier, and microbiota of LPS challenge broilers via the LPS challenge immune stress model.

## 2. Materials and Methods

### 2.1. Experimental Design

A total of 288 healthy one-day-old yellow-feathered male broilers (32.94 g ± 0.10 g, Tang Renshen Group Co., Ltd., Zhuzhou, China) were randomly assigned to 4 groups, including the CON group (basal diet + saline), the LPS group (basal diet+ LPS), the SF group (basal diet + 1000 mg/kg SF + saline), and the SF + LPS group (basal diet + 1000 mg/kg SF + LPS), with 6 replicates of 12 broilers each. SF (purity ≥ 30%) was provided by Baoji Fangsheng Biological Development Co., Ltd. (Xi’an, China) and fed from 1 day of age; the dose of SF supplemented was referred to in previous studies [19]. LPS (*Escherichia coli* O55: L2880) was obtained from Sigma Aldrich (St. Louis, MO, USA). LPS was dissolved in saline, and 500 μg LPS/mL was prepared before use. LPS-challenged broilers were intraperitoneally injected with 500 μg LPS /kg on 16, 18, and 20 d; non-LPS-challenged broilers were injected with an equal amount of saline, and the frequency and dosage of LPS administration were selected based on previous studies [20,21]. The feeding trial lasted for 20 days. The basal diet (Table 1) was formulated based on the feeding standards of chickens (NY/T33-2004). All broilers were housed on the net floor (cage size: 2.5 m × 1.5 m), which had free access to feed and water. The artificial continuous lighting system was used, with temperatures ranging from 33 °C to 34 °C in the first week and 27 °C to 31 °C in the second week. Heating was applied depending on the specific temperature conditions. Natural ventilation in chicken coops. Maintain a relative humidity between 55 and 65%.

### 2.2. Sample Collection

At the age of 21 days, one healthy bird with similar weight was selected from each replicate (fasting 8–12 h in advance, and live weight was recorded) and euthanized. Blood was collected using common tubes (Liuyang Sanli Medical Science and Technology Development Co., Ltd., Liuyang, China), centrifuged at 3500 r /min at 4 °C for 10 min, taken as the supernatant, and stored in −20 °C freezers. Open the abdominal cavity; the whole intestinal segment was removed and separated (duodenum, jejunum, and ileum). The middle segment was taken about 2 cm and fixed in 4% paraformaldehyde solution (Wuhan Kanos Technology Co., Ltd., Wuhan, China), scraped portions of the mucosa, and stored in −80 °C freezers. Appropriate amounts of cecal chyme were collected and stored in the refrigerator at −80 °C.

### 2.3. Index Determination Method

#### 2.3.1. Measurement of Growth Performance

Initial weight (IW), final weight (FW), and feed consumption of each cage were recorded to calculate the average daily gain (ADG), average daily feed intake (ADFI), and feed-to-gain ratio (FCR) in the non-LPS challenged period and LPS challenged period.

#### 2.3.2. Determination of Serum Indicators

The concentrations of interleukin-1β (IL-1β), interleukin-2 (IL-2), interleukin-10 (IL-10), tumor necrosis factor-α (TNF-α), corticosterone (CORT), D-lactate (D-LA), and endotoxin were determined by ELISA kits. The kits were purchased from Jiangsu Enzyme-Free Industrial Co., Ltd. (Yancheng, China), as described by Cheng et al. [22].

#### 2.3.3. Gut Histomorphological Analysis

The duodenum, jejunum, and ileum samples were removed from a 4% paraformaldehyde fixing solution, dehydrated and embedded with paraffin, and cut into 5 μm slices, as described by Chang et al. [23]. Villi height (VH) and crypt depth (CD) were measured by optical microscopy in combination with software (Image Analysis 1.6.1) after staining with hematoxylin eosin (HE), magnifying 40×, and calculating the ratio of villus height to crypt depth (V/C) from the results. Six good villi and crypts were selected from each section for measurement, and the average value was taken.

#### 2.3.4. Gene Expression Using Quantitative Real-Time PCR

Total RNA was extracted from jejunal mucosal tissue samples using AG reagents (Hangzhou Aikerui Biotechnology Co., Ltd., Hangzhou, China). RNA integrity was evaluated by agarose gel. and RNA concentration and purity were measured by a Nanodrop ND-2000 spectrophotometer (Thermo Scientific, Ottawa, ON, Canada). Reverse transcribed into cDNA using AG reagents (Hangzhou Aikerui Biotechnology Co., Ltd., Hangzhou, China). All primers (Table 2) were purchased from Changsha Biotechnology Co., Ltd. Real-time quantitative PCR detection by the SYBR Green PCR kit (Hangzhou Aikerui Biotechnology Co., Ltd., Hangzhou, China) in CFX Connect real-time PCR detection system, measured β-actin, Zonula occludens-1 (ZO-1), Occludin, Claudin-1, MUC-2, Toll-like receptor 4 (TLR4), myeloid differentiation primary response 88 (MyD88), and nuclear factor kappa B (NF-κB) relative mRNA expression levels. Results were calculated using the 2^−ΔΔCT^ method, as described by Xing et al. [24].

#### 2.3.5. 16S rRNA Sequencing

Genomic DNA was extracted using a commercial kit (Qiagen, Hilden, Germany). The 16S rRNA was amplified by 338F (ACTCCTACGGGAGGCAGCAG) and 806R (GGACTACHVGGGTWTCTAAT) primers, then sequenced using Illumina. Bioinformatics analysis was performed using the online platform (https://cloud.majorbio.com, accessed on 11 September 2023) provided by Shanghai Majorbio Bio-Pharm Technology Co. Ltd. (Shanghai, China).

### 2.4. Statistical Analysis

All data were carried out using the Shapiro-Wilk test, analyzed by two-way ANOVA, and then Duncan multiple comparisons were performed with IBM SPSS statistical software (26.0 version, Chicago, IL, USA). The main effects include the LPS challenge and SF treatment, as well as the interactions between LPS and SF. Data were expressed as mean ± standard error of the mean (SEM). Values of *p* < 0.05 were regarded as statistically remarkable.

## 3. Results

### 3.1. Growth Performance

The growth performance results were presented in Table 3. There was no remarkable difference in growth performance between broilers fed with SF and without SF (*p* > 0.05) before the LPS challenge (1–15 days old). However, LPS lowered ADG, ADFI, and FW (*p* < 0.05) and increased FCR (*p* < 0.05) during the LPS challenge (16–20 days old) when compared with that of the control. SF supplementation significantly enhanced ADG and FW (*p* < 0.05), and the ADFI presented a tendency to increase (0.05 < *p* < 0.1), but the FCR had no significant effect (*p* > 0.05). However, there was no remarkable interaction between SF and LPS on the growth performance of broilers (*p* > 0.05). The details were presented in Table 4.

### 3.2. Serum Inflammation

**Figure 1:**
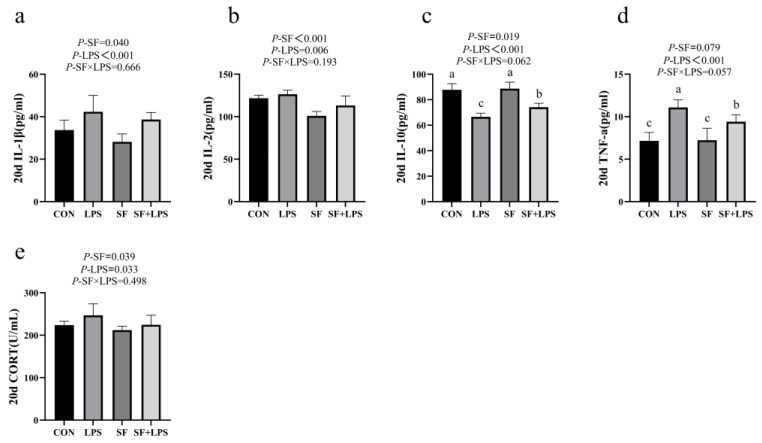
Serum inflammation and immune indexes in 20 d broilers (**a**–**e**). Data represent mean values of six replicates per group (*n* = 6). ^a–c^ Means in the same row with different superscripts differ (*p* < 0.05). CON: basal diet + saline; LPS: basal diet +500 μg/kg LPS; SF: 1000 mg/kg SF + saline; SF + LPS: 1000 mg/kg SF + 500 μg/kg LPS.

As presented in Figure 1, the LPS challenge remarkably elevated the serum concentration of IL-1β, IL-2, TNF-α, and CORT (*p* < 0.05) and decreased the concentration of IL-10 (*p* < 0.05) when compared with that of the control. SF supplementation markedly attenuated IL-1β, IL-2, and CORT concentrations and increased IL-10 concentrations (*p* < 0.05). In addition, the multiple comparisons show that the LPS group had a significantly lower serum concentration of IL-10 and a higher concentration of TNF-α compared with the SF + LPS group (*p* < 0.05).

### 3.3. Intestinal Morphology

**Figure 2:**
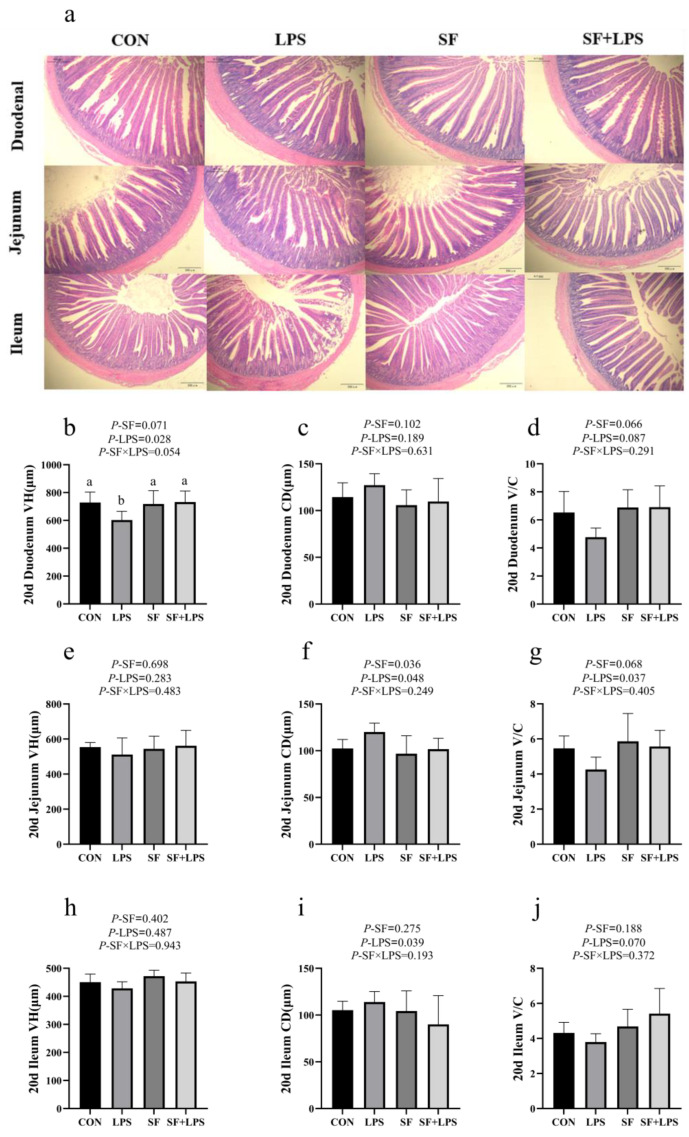
Histological morphology of duodenum, jejunum, and ileum in 20 d broilers (**a**–**j**). Data represent mean values of six replicates per group (*n* = 6). ^ab^ Means in the same row with different superscripts differ (*p* < 0.05). CON: basal diet + saline; LPS: basal diet +500 μg/kg LPS; SF: 1000 mg/kg SF + saline; SF + LPS: 1000 mg/kg SF + 500 μg/kg LPS.

Severe damage to the intestinal morphology of broilers was observed by the LPS challenge (Figure 2a). As shown in Figure 2, LPS challenge significantly lowered duodenum VH and jejunum V/C (*p* < 0.05) compared with that of control broilers, while an opposite trend was observed for jejunum and ileum CD (*p* < 0.05) and tended to lower duodenum and ileum V/C (0.05 < *p* < 0.1). SF addition significantly reduced jejunal CD (*p* < 0.05) and elevated duodenal VH, V/C, and jejunal V/C (0.05 < *p* < 0.1). Multiple comparisons have shown that the duodenal VH of the SF + LPS group was significantly higher than that of the LPS group (*p* < 0.05). SF supplementation could alleviate these negative effects of broilers in duodenal VH stimulated by LPS.

### 3.4. Intestinal Permeability and Expression of Jejunal Mucosa-Associated Gene mRNA

**Figure 3:**
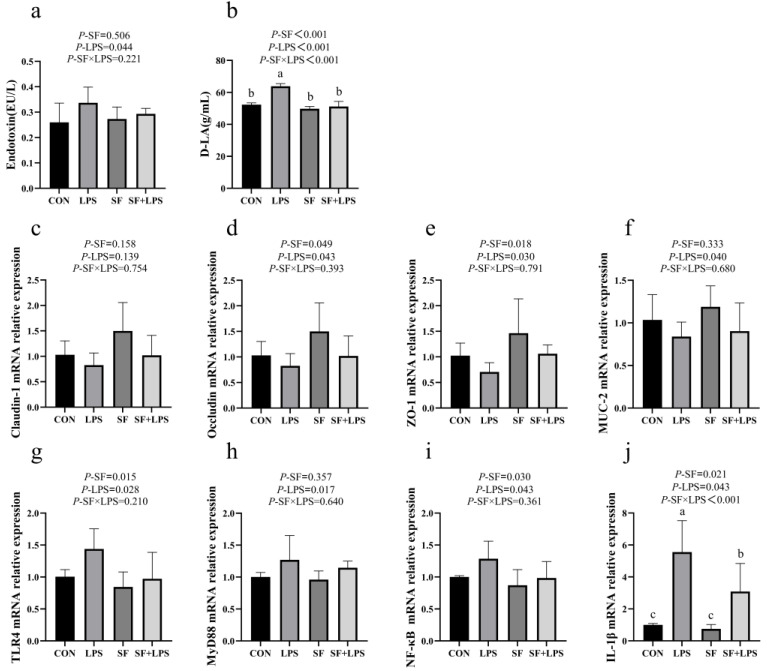
Intestinal permeability and relative expression of jejunal mucosa-associated gene mRNA in 20 d broilers (**a**–**j**). Data represent mean values of six replicates per group (*n* = 6). ^a–c^ Means in the same row with different superscripts differ (*p* < 0.05). CON: basal diet + saline; LPS: basal diet +500 μg/kg LPS; SF: 1000 mg/kg SF + saline; SF + LPS: 1000 mg/kg SF + 500 μg/kg LPS.

As shown in Figure 3, LPS stress significantly increased the endotoxin, D-LA levels in serum, and TLR4, MyD88, NF-κB, and IL-1β relative gene expression in the jejunal mucosa (*p* < 0.05) when compared with that of the control, and signally decreased the MUC-2, Occludin, and ZO-1 relative gene expression in the jejunal mucosa in broilers (*p* < 0.05). On the contrary, SF supplementation reduced D-LA level and TLR4, NF-κB, and IL-1β relative gene expression in broilers (*p* < 0.05) and increased the relative expression of Occludin and ZO-1. However, no major changes in Claudin-1, MUC-2, or MyD88 mRNA expression in the jejunal mucosa were observed after SF supplementation (*p* > 0.05). There was a remarkable interaction between SF and LPS on D-LA level and IL-1β mRNA expression (*p* < 0.05). Multiple comparisons have shown that the relative expression levels of D-LA and IL-1β mRNA in the SF + LPS group were significantly lower than those in the LPS group (*p* < 0.05). SF supplementation could reverse these adverse effects in the serum D-LA level and jejunal mucosa IL-1β relative mRNA expression of broilers stimulated by LPS.

### 3.5. Gut Microbiota Analysis

**Figure 4:**
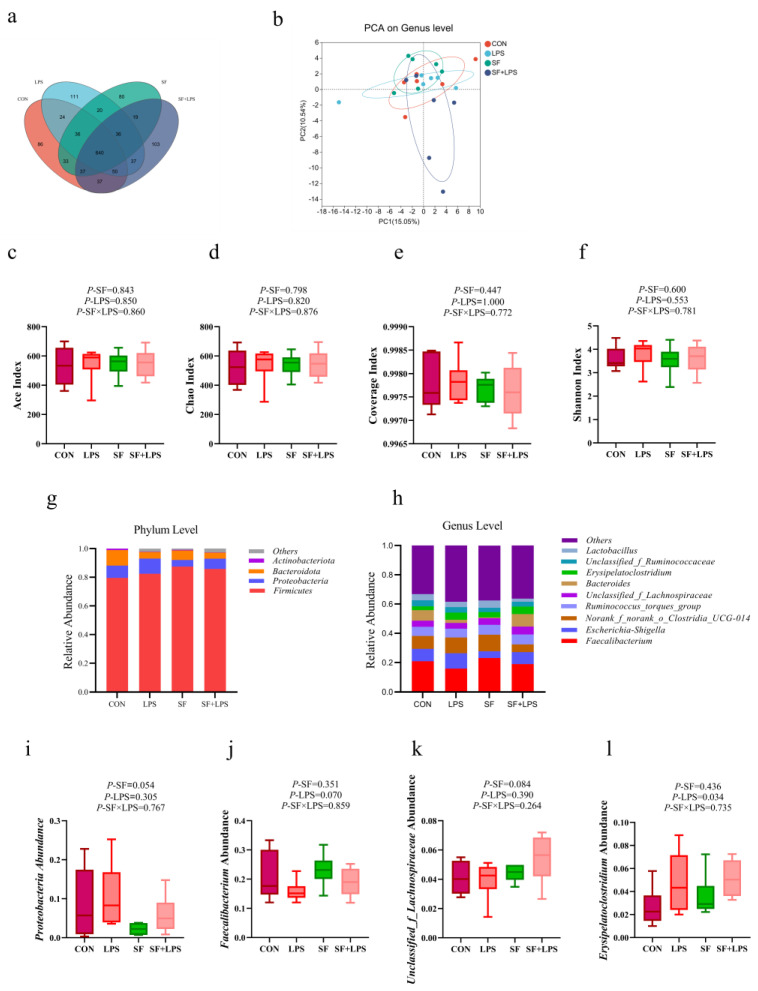
Venn diagram (**a**). Alpha diversity of cecal flora (**b**–**e**). Principal component analysis (PCA) of cecal flora (**f**). The relative abundance of cecal microbiota composition at the phylum and genus levels (**g**–**l**). CON: basal diet + saline; LPS: basal diet +500 μg/kg LPS; SF: 1000 mg/kg SF + saline; SF + LPS: 1000 mg/kg SF + 500 μg/kg LPS.

**Figure 5:**
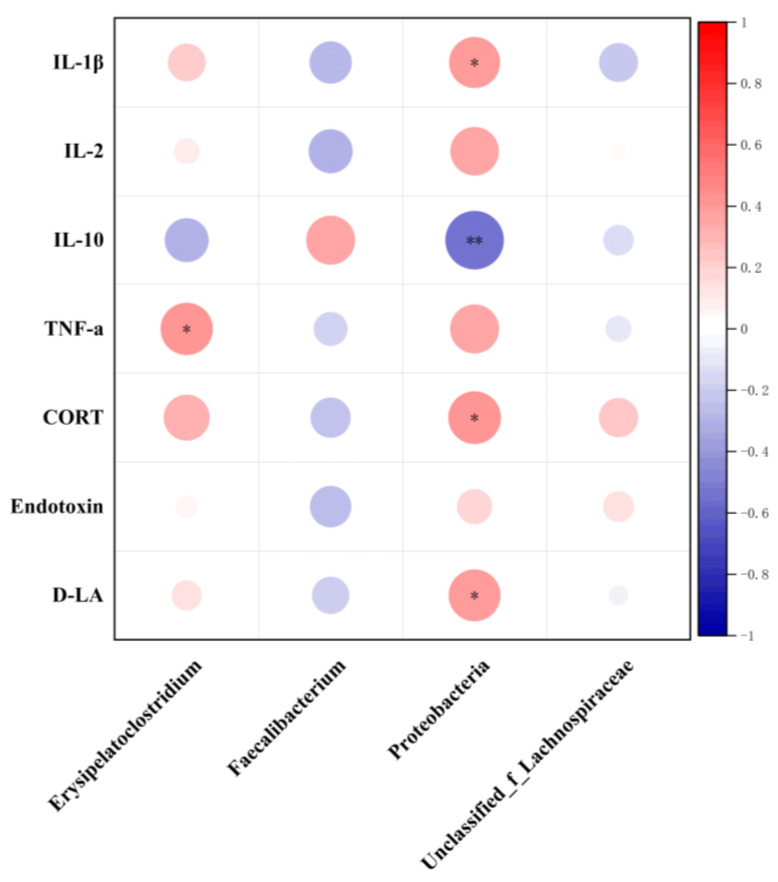
Correlation analysis of cecal flora and inflammation indexes. Data represent mean values of six replicates per group (*n* = 6). * Indicates *p* < 0.05. ** Indicates *p* < 0.01.

At the 97% similarity level, 969,264 high-throughput sequence reads were obtained from all cecal content samples (*n* = 6), with an average of 40,386 sequences per sample. There were 640 core OTUs shared by the cecal flora in each group, and there were 943 OTUs in the cecal flora of the CON group, 954 OTUs in the cecal flora of the LPS group, 901 OTUs in the cecal flora of the SF group, and 959 OTUs in the cecal flora of the SF + LPS group (Figure 4a). No significant difference in community richness of cecal microbiota was observed in broilers, including Ace, Chao, Shannon, Coverage, and Simpson (*p* > 0.05, Figure 4c–f). The PCA map based on the genus level shows no difference between the four groups of cecal flora (Figure 4b). At the phylum level, the cecal flora consisted mainly of Firmicutes, Proteobacteria, Bacteroidota, and Actinobacteriota in broilers (Figure 4g). Firmicutes, Proteobacteria, and Bacteroidota were the dominant phyla, and their relative abundances were analyzed. SF supplementation presented a tendency to decrease the relative abundance of proteobacteria (0.05 < *p* < 0.1, Figure 4i). At the genus level, the cecal flora consisted mainly of *Faecalibacterium*, *Escherichia-Shigella*, Norank_f_norank_o_Clostridia_UCG-014, *Ruminococcus_torques*_group, Unclassified_f_Lachnospiraceae, *Bacteroides*, *Erysipelatoclostridium*, Unclassified_f_Ruminococcaceae, and *Lactobacillus* in broilers (Figure 4h). Compared with nonchallenged broilers, LPS presented a tendency to lower the relative abundance of *Faecalibacterium* (0.05 < *p* < 0.1, Figure 4j) and elevate the relative abundance of *Erysipelatoclostridium* (*p* < 0.05, Figure 4l). In addition, SF supplementation presented a tendency to increase the relative abundance of Unclassified_f_Lachnospiraceae (0.05 < *p* < 0.1, Figure 4k). Spearman correlation analysis confirmed that changes in intestinal flora were associated with LPS treatment and SF supplementation (Figure 5). There was an obviously negative correlation between IL-10 and Proteobacteria (*p* < 0.05), and a positive correlation between IL-1β, CORT, D-LA, and Proteobacteria (*p* < 0.05). In addition, TNF-α and *Erysipelatoclostridium* were positively correlated (*p* < 0.05).

## 4. Discussion

CORT serves as a crucial indicator of hypothalamus–pituitary–adrenal axis activity. Elevated CORT levels directly inhibit growth under immune stress conditions in broilers [25]. In our study, the CORT level of broilers was significantly increased after injection of LPS, validating successful modeling for subsequent tests. In a similar experiment, SF supplementation markedly enhanced the ADG and FW of broilers at days 21, 42, and played a role in improving nutrient digestion and absorption [26]. Quercetin, the primary active component of SF, upregulated the expression of nutrient transporter genes in the intestine, such as glucose transporter 2 and peptide transporter 1, promoted intestinal digestion and absorption, improved intestinal morphology, protected the intestinal barrier, and improved growth performance [27]. Dietary incorporation of 1000 mg/kg SF significantly influenced ADG and FW in broilers, which may be closely related to its active components. The utilization rate of feed and the digestion and absorption of nutrients are critical factors influencing growth performance, especially in the intestine, which plays an important role as the main site for the digestion and absorption of nutrients [28]. The specific reasons need to be further studied.

Intestinal morphology is a key indicator for evaluating digestion [29]. It is primarily characterized by VH, CD, and V/C indexes; longer VH and shallower CD can provide a larger area of intestinal nutrient absorption, on the contrary, which may cause intestinal inflammation and impair intestinal function. Therefore, greater V/C signifies higher absorption capacity; a normal intestinal structure is essential for growth and development [30]. In this study, it was observed that LPS stimulation significantly impaired the intestinal morphology of broilers, which aligns with previous research findings [31,32]. Notably, dietary SF supplementation alleviated the adverse effects of LPS on intestinal morphology by reducing jejunal CD and increasing duodenal VH as well as V/C and jejunal V/C ratios. Thus, SF supplementation-enhanced intestinal VH and V/C may improve intestinal morphology by promoting the proliferation and differentiation of intestinal epithelial cells while increasing the surface area for nutrient absorption in the intestine [33].

D-LA and endotoxin are commonly utilized for evaluating intestinal permeability and intestinal mucosa damage. D-LA and endotoxin are the byproducts of lactic acid fermentation and cleavage of bacteria in the intestine, exclusive to this tissue [34,35]. Under normal conditions, levels of D-LA and endotoxin in the blood of livestock and poultry are minimal, increasing only when there is intestinal mucosal damage [36,37]. Increased intestinal permeability due to damaged integrity leads to decreased nutrient digestion and absorption in livestock and poultry, impacting poultry growth performance [38]. Our study also revealed that intraperitoneal injection of LPS raised the levels of D-LA and endotoxin in broilers, indicating LPS-induced damage to the broiler’s intestinal mucosa barrier function. However, SF supplementation significantly reduced the D-LA level in broilers. These results suggest that SF mitigated LPS-induced impairment of broiler intestinal permeability while positively influencing their mucosal barrier.

The tight junction is the crucial connection between intestinal epithelial cells. Tight junction proteins are essential components of the intestinal barrier protein, playing a crucial role in its composition and function [39]. Numerous studies have shown that during an inflammatory response, various inflammatory factors enter the intestine, leading to intestinal lesions, damage to the intestinal tissue, disruption of the barrier function, and reducing the Occludin, ZO-1, and Claudin-1 mRNA expression levels [40,41]. In this experiment, LPS stimulation significantly decreased the relative gene expression of MUC-2, Occludin, and ZO-1 in the jejunum mucosa of broilers. The expression of intestinal mucosal barrier genes in broilers was lowered, and intestinal permeability was impaired by intraperitoneal injection of LPS in broilers, which was also supported by elevated serum D-LA and endotoxin. It has been reported that dietary supplementation with flavonoids up-regulates the expression of tight junction proteins (especially ZO-1, Claudin-1, E-cadherin, and Occludin) in broilers at days 21 and provides protective benefit against LPS-challenged dysfunction in their intestinal mucosal barriers [42]. The study yielded similar findings: dietary SF could effectively up-regulate the relative gene expression of Occludin and ZO-1 in the jejunum mucosal of broilers. In summary, we recommend that SF supplementation at 1000 mg/kg enhances the expression of tight junctions in the small intestine challenged by LPS and maintains the integrity of the intestinal mucosal barrier.

When poultry is exposed to LPS, LPS binds to TLR4 and initiates a cascade reaction that stimulates the TLR4/NF-κB signaling pathway, leading to the secretion of a number of pro-inflammatory cytokines (IL-1β, IL-2, IL-6, TNF-α, et al.) by monocytes and macrophages, resulting in tissue damage [43]. NF-κB plays a crucial role in activating inflammation through the synthesis and release of pro-inflammatory cytokines [44,45]. Consistent with previous findings, this study observed that LPS significantly elevated serum inflammatory, and up-regulated the expression of genes related to the TLR4/NF-κB signaling pathway in the jejunum mucosa, confirming the LPS-mediated inflammatory response. It has been reported that flavonoids can reduce the occurrence of disease by negatively regulating the inflammatory factor IL-1β [46]. Quercetin, kaempferol, and isorhamnetin are the main active components of SF, which can enhance animal immunity and reduce inflammation [47]. SF shows great potential for treating inflammation, as does quercetin [48] and kaempferol [49]. and isorhamnetin [50] can inhibit the activation of the TLR4/NF-κB signaling pathway, thereby suppressing the production of pro-inflammatory cytokines. The results demonstrated that the addition of SF significantly reduced the levels of IL-1β, IL-2, and CORT in serum, as well as the relative expression levels of TLR4, NF-κB, and IL-1β mRNA in the jejunum mucosa. Additionally, it markedly elevated the concentration of IL-10 in serum. The results indicated that the anti-inflammatory effect of SF was closely related to the TLR4/NF-κB signaling pathway.

Intestinal flora is a complex ecosystem that plays a vital role in the digestion and absorption of nutrients, metabolism, immune regulation, and other processes. Numerous studies have demonstrated the vital importance of intestinal flora in maintaining overall body health [51]. Intestinal microbes can regulate the whole immune system, strengthen the intestinal barrier function, and thus improve the production performance of poultry [52]. It has been reported that the abundant proteobacteria could easily lead to bacterial imbalance and inflammatory responses [53]. The relative abundance of proteobacteria in mice significantly increased after long-term mildronate treatment, leading to a bacterial imbalance [54]. Xie et al. [55] found that quercetin can improve the intestinal environment and down-regulate the relative abundance of proteobacteria and other microorganisms. Supplementation with SF reduced the relative abundance of proteobacteria, which is basically consistent with previous studies. Furthermore, Spearman correlation analysis also revealed that IL-10 was significantly negatively correlated with Proteobacteria, while IL-1β, CORT, and D-LA were significantly positively correlated with Proteobacteria, indicating that Proteobacteria increased inflammation and reduced immune response. *Faecalibacterium* is a crucial intestinal bacterium with various probiotic effects, such as maintaining intestinal homeostasis and anti-inflammatory properties [56]. LPS challenge enriched potentially harmful microbes and led to an increase in *Erysipelatoclostridium*, which is indicative of intestinal disorders causing inflammation [57]. According to the study, Fuzhuan brick tea crude polysaccharides (FBTPs) restored the microbial imbalance induced by cyclophosphamide (Cy) by elevating several beneficial bacteria, such as lactic acid bacteria, Unclassified_f_Lachnospiraceae, while reducing Bacteroides and *Helicobacter pylori*, thus playing an immune protective role in Cy-induced mice [58]. In this experiment, LPS lowered the relative abundance of *Faecalibacterium* and elevated the relative abundance of *Erysipelatoclostridium*. In addition, SF supplementation presented a tendency to increase the relative abundance of Unclassified_f_Lachnospiraceae. In addition, Spearman correlation analysis showed that TNF-α and *Erysipelatoclostridium* were positively correlated, which supported this point. Therefore, we found that SF can up-regulate the relative abundance of beneficial bacteria and down-regulate the relative abundance of harmful bacteria, maintain intestinal health, and improve the production performance of broilers.

## 5. Conclusions

In conclusion, dietary supplementation with 1000 mg/kg of SF could enhance the growth performance of broilers by mitigating the serum inflammatory response, improving intestinal health via enhancing intestinal morphology and regulating the intestinal barrier, and effectively alleviating the damage caused by LPS.

## Figures and Tables

**Table 1:** Composition and nutrient levels of basic diet (air dried basis, %).

Ingredients, %	Content	Nutrient Levels, % ^2^	Content
Corn	56.78	Metabolizable energy, MJ/kg	2879
Soybean meal	29.00	Crude protein	20.06
Corn gluten meal	5.00	Calcium	0.921
Wheat bran	3.60	Phosphorus	0.55
Soybean oil	1.20	Lysine	1.20
CaHPO_4_	1.30	Methionine	0.54
Limestone	1.20	Methionine + Cysteine	0.88
Sodium chloride	0.30	Threonine	0.77
L-Lysine	0.54	Tryptophan	0.21
DL-Methionine	0.18		
Choline (60%)	0.10		
Premix ^1^	0.80		
Total	100		

^1^ Premix provided the following per kg of the diet: vitamin A 8000 IU, vitamin D3 4000 IU, vitamin E 15 IU, vitamin K 15 mg, vitamin B1 2.0 mg, vitamin B2 4.0 mg, vitamin B6 3.0 mg, vitamin B12 0.015 mg, folic acid 0.9 mg, nicotinic acid 35 mg, pantothenic acid 15 mg, biotin 0.2 mg, Mn 90 mg, Fe 90 mg, Zn 60 mg, Cu 10 mg, Se 0.20 mg, and I 0.40 mg. ^2^ The content of crude protein, calcium, and total phosphorus in the basic dietary nutrition level are all measured values, while the rest are calculated values.

**Table 2:** Real time PCR primer sequences.

Genes	Primer ^1^	Sequence	Product Size (bp)	GenBank Accession Number
*Occludin*	F:5′-3′	GATGGACAGCATCAACGACC	142	NM_205128.1
	R:5′-3′	CTTGCTTTGGTAGTCTGGGC		
*Claudin-1*	F:5′-3′	ACACCCGTTAACACCAGATTT	152	NM_001013611.2
	R:5′-3′	GCATTTTTGGGGTAGCCTCG		
*ZO-1*	F:5′-3′	GCCTACTGCTGCTCCTTACAACTC	129	XM_040680630.1
	R:5′-3′	GCTGGATCTATATGCGGCGGTAAG		
*MUC-2*	F:5′-3′	GTGAATGGCACTACGAGCCT	106	XM_040701656.2
	R:5′-3′	CTGGGGTAGCAACCTTCCAG		
*TLR4*	F:5′-3′	TGGATCTTTCAAGGTGCCACA	198	NM_001030693.2
	R:5′-3′	AGTGTCCGATGGGTAGGTCA		
*NF-κB*	F:5′-3′	TCAACGCAGGACCTAAAGACAT	162	NM_205134.1
	R:5′-3′	GCAGATAGCCAAGTTCAGGATG		
*MyD88*	F:5′-3′	GGATGATCCGTATGGGCATGG	171	NM_001030962.5
	R:5′-3′	ATGGACCACACACACGTTCC		
*IL-1β*	F:5′-3′	ACTGGGCA TCAAGGGCTA	154	NM_214005.1
	R:5′-3′	GGTAGAAGA TGAAGCGGGTC		
*β-actin*	F:5′-3′	TGCGTGACATCAAGGAGAAG	199	L08165
	R:5′-3′	TGCCAGGGTACATTGTGGTA		

^1^ F = forward primer; R = reverse primer.

**Table 3:** Effects of sea-buckthorn flavonoids supplementation on growth performance of broilers ^1^.

Items ^2^	Group ^3^		SEM ^4^	*p*-Value
	CON	SF		
Before LPS challenge (1 to 15 day of age)				
IW (g)	32.93	32.96	0.069	0.968
ADG (g)	15.70	15.73	0.177	0.561
ADFI (g)	25.07	25.17	0.179	0.826
FCR	1.60	1.61	0.014	0.175
FW (g)	268.43	266.35	2.556	0.602

^1^ Data represent mean values of 12 replicates per group (*n* = 12). ^2^ IW: initial weight; ADG: average daily gain; ADFI: average daily feed intake; FCR: feed-to-gain ratio ^3^ CON: basal diet; SF: basal diet +1000 mg/kg SF. ^4^ SEM: standard error of the mean.

**Table 4:** Effects of sea-buckthorn flavonoids supplementation on the growth performance of lipopolysaccharide-challenged broilers ^1^.

Items ^2^	Group ^3^				SEM ^4^	*p*-Value		
	CON	LPS	SF	SF + LPS		SF	LPS	SF × LPS
During LPS challenge (16 to 20 days of age)								
ADG(g)	26.58	23.43	28.03	25.58	0.759	0.028	0.001	0.652
ADFI(g)	49.90	47.54	51.20	50.31	0.882	0.079	0.032	0.414
FCR	1.88	2.04	1.83	1.97	0.062	0.346	0.022	0.878
FW(g)	400.26	386.61	404.10	396.68	3.177	0.041	0.003	0.338

^1^ Data represent mean values of six replicates per group (*n* = 6). ^2^ IW: initial weight; ADG: average daily gain; ADFI: average daily feed intake; FCR: feed-to-gain ratio ^3^ CON: basal diet + saline; LPS: basal diet +500 μg/kg LPS; SF: 1000 mg/kg SF + saline; SF + LPS: 1000 mg/kg SF + 500 μg/kg LPS. ^4^ SEM: standard error of the mean.

## Data Availability

All datasets collected and analyzed during the current study are available from the corresponding author by request, the availability of the data is restricted to investigators based at academic institutions.

## References

[1] Gao L.A., Er M.W., Li L.H., Wen P., Jia Y.C., Huo L.M. (2022). Microclimate environment model construction and control strategy of enclosed laying brooder house. Poult Sci..

[2] Liu Y.H., Zhang Y., Bai D.Y., Li Y.Q., He X.L., Ito K., Liu K., Tan H., Zhen W., Zhang B. (2023). Dietary Supplementation with Chlorogenic Acid Enhances Antioxidant Capacity, Which Promotes Growth, Jejunum Barrier Function, and Cecum Microbiota in Broilers under High Stocking Density Stress. Animals.

[3] Yao M., Gao W.H., Tao H.X., Yang J., Huang T.H. (2017). The regulation effects of danofloxacin on pig immune stress induced by LPS. Res. Vet. Sci..

[4] Wang M.Y., Zhang Y., Tong Y.X., Guo P.T., Zhang J., Wang C.K., Gao Y.Y. (2022). Effects of lutein on jejunal mucosal barrier function and inflammatory responses in lipopolysaccharide-challenged yellow-feather broilers. Poult Sci..

[5] Dias K.M.M., Oliveira C.H., Calderano A.A., Rostagno H.S., Gomes K.M., O’Connor K.E., Davis R., Walsh M., Britton J., Altieri E.A. (2024). Dietary Hydroxytyrosol Supplementation on Growth Performance, Gut Morphometry, and Oxidative and Inflammatory Status in LPS-Challenged Broilers. Animals.

[6] Ding M., Yu Y., Zhu Z., Tian H., Guo Y., Zan R., Tian Y., Jiang R., Li K., Sun G. (2023). Regulation of the MyD88 gene in chicken spleen inflammation induced by stress. J. Anim. Sci..

[7] Yang Z., Yang J.J., Zhu P.J., Han H.M., Wan X.L., Yang H.M., Wang Z.Y. (2022). Effects of betaine on growth performance, intestinal health, and immune response of goslings challenged with lipopolysaccharide. Poult Sci..

[8] Ciesarová Z., Murkovic M., Cejpek K., Kreps F., Tobolková B., Koplík R., Belajová E., Kukurová K., Daško Ľ., Panovská Z. (2020). Why is sea buckthorn (*Hippophae rhamnoides* L.) so exceptional? A review. Food Res Int..

[9] Sireswar S., Biswas S., Dey G. (2020). Adhesion and anti-inflammatory potential of Lactobacillus rhamnosus GG in a sea buckthorn based beverage matrix. Food Funct..

[10] Sławińska N., Żuchowski J., Stochmal A., Olas B. (2023). Extract from Sea Buckthorn Seeds-A Phytochemical, Antioxidant, and Hemostasis Study; Effect of Thermal Processing on Its Chemical Content and Biological Activity In Vitro. Nutrients.

[11] Jayashankar B., Singh D., Tanwar H., Mishra K.P., Murthy S., Chanda S., Mishra J., Tulswani R., Misra K., Singh S.B. (2017). Augmentation of humoral and cellular immunity in response to Tetanus and Diphtheria toxoids by supercritical carbon dioxide extracts of *Hippophae rhamnoides* L. leaves. Int. Immunopharmacol..

[12] Xiao P.T., Liu S.Y., Kuang Y.J., Jiang Z.M., Lin Y., Xie Z.S., Liu E.H. (2021). Network pharmacology analysis and experimental validation to explore the mechanism of sea buckthorn flavonoids on hyperlipidemia. J Ethnopharmacol..

[13] Sharma A., Shukla P.K., Bhattacharyya A., Kumar U., Roy D., Yadav B., Prakash A. (2018). Effect of dietary supplementation of sea buckthorn and giloe leaf meal on the body weight gain, feed conversion ratio, biochemical attributes, and meat composition of turkey poults. Vet. World.

[14] Hao X.Y., Zhang X.Z., Yang D.Y., Xie Y.Z., Mu C.T., Zhang J.X. (2023). Effects of sea-buckthorn flavonoids on growth performance, nutrient digestibility, microbial protein synthesis, and plasma antioxidant capacity of finishing lambs. Anim. Feed. Sci. Technol..

[15] Ren Q.C., Li X.H., Li Q.Y., Yang H.L., Wang H.L., Zhang H., Zhao L., Jiang-Yong S.L., Meng X.L., Zhang Y. (2019). Total flavonoids from sea buckthorn ameliorates lipopolysaccharide/cigarette smoke-induced airway inflammation. Phytother Res..

[16] Xu Y., Li J., Lin Z., Liang W., Qin L., Ding J., Chen S., Zhou L. (2022). Isorhamnetin Alleviates Airway Inflammation by Regulating the Nrf2/Keap1 Pathway in a Mouse Model of COPD. Front Pharmacol..

[17] Sul O.J., Ra S.W. (2021). Quercetin Prevents LPS-Induced Oxidative Stress and Inflammation by Modulating NOX2/ROS/NF-kB in Lung Epithelial Cells. Molecules.

[18] Bian Y., Lei J., Zhong J., Wang B., Wan Y., Li J., Liao C., He Y., Liu Z., Ito K. (2022). Kaempferol reduces obesity, prevents intestinal inflammation, and modulates gut microbiota in high-fat diet mice. J. Nutr. Biochem..

[19] Ma J.S., Chang W.H., Liu G.H., Zhang S., Zheng A.J., Li Y., Xie Q., Liu Z.Y., Cai H.Y. (2015). Effects of flavones of sea buckthorn fruits on growth performance, carcass quality, fat deposition and lipometabolism for broilers. Poult Sci..

[20] An J.S., Shi J.J., Liu K.B., Li A.K., He B.B., Wang Y., He J.H. (2022). Effects of Solid-State Fermented Wheat Bran on Growth Performance, Immune Function, Intestinal Morphology and Microflora in Lipopolysaccharide-Challenged Broiler Chickens. Animals.

[21] Zhang P.F., Shi B.L., Su J.L., Yue Y.X., Cao Z.X., Chu W.B., Li K., Yan S.M. (2017). Relieving effect of Artemisia argyi aqueous extract on immune stress in broilers. J. Anim. Physiol. Anim. Nutr..

[22] Cheng Y., Liu S., Wang F., Wang T., Yin L., Chen J., Fu C. (2024). Effects of Dietary Terminalia chebula Extract on Growth Performance, Immune Function, Antioxidant Capacity, and Intestinal Health of Broilers. Animals.

[23] Chang W.Y., Yu Y.H. (2022). Effect of Bacillus species-fermented products and essential oils on growth performance, gut morphology, cecal short-chain fatty acid levels, and microbiota community in broilers. Poult. Sci..

[24] Xing Y.Y., Zheng Y.K., Yang S., Zhang L.H., Guo S.W., Shi L.L., Xu Y.Q., Jin X., Yan S.M., Shi B.L. (2021). Artemisia ordosica Polysaccharide Alleviated Lipopolysaccharide-induced Oxidative Stress of Broilers via Nrf2/Keap1 and TLR4/NF-κB Pathway. Ecotoxicol. Environ. Saf..

[25] Li X., Bian J., Xing T., Zhao L., Li J.L., Zhang L., Gao F. (2023). Effects of guanidinoacetic acid supplementation on growth performance, hypothalamus-pituitary-adrenal axis, and immunity of broilers challenged with chronic heat stress. Poult Sci..

[26] Kalia S., Bharti V.K., Giri A., Kumar B., Arora A., Balaje S.S. (2018). *Hippophae rhamnoides* as novel phytogenic feed additive for broiler chickens at high altitude cold desert. Sci Rep..

[27] Abdel-Latif M.A., Elbestawy A.R., El-Far A.H., Noreldin A.E., Emam M., Baty R.S., Albadrani G.M., Abdel-Daim M.M., Abd El-Hamid H.S. (2021). Quercetin Dietary Supplementation Advances Growth Performance, Gut Microbiota, and Intestinal mRNA Expression Genes in Broiler Chickens. Animals.

[28] Liu X., Huang X.Y., Fu Y., Wang Y.Z., Lu Z.Q. (2022). The Positive Effects of Exogenous Pancreatin on Growth Performance, Nutrient Digestion and Absorption, and Intestinal Microbiota in Piglets. Front. Physiol..

[29] Song X., Anas M.A., Kurniawati A., Hanim C., Muhlisin A.M.A., Madani A.M.A., Wang Q., Chen H. (2023). Effects of reduced-protein diets with protease supplementation on growth, carcass yield, intestinal morphology, organ development, nutrient digestibility, and blood biochemical of broiler chickens. Transl. Anim. Sci..

[30] Zou T.D., Deng C.X., Wang Z.R., Ye Y.L., You J.M. (2019). Dietary alanyl-glutamine improves growth performance of weaned piglets through maintaining intestinal morphology and digestion-absorption function. Animal.

[31] Xing Y., Zheng Y., Yang S., Zhang L., Guo S., Shi L., Xu Y., Jin X., Yan S., Shi B.L. (2023). Artemisia ordosica polysaccharide ameliorated LPS-induced growth inhibition and intestinal injury in broilers through enhancing immune-regulation and antioxidant capacity. J. Nutr. Biochem..

[32] Sun H., Zheng X., Yang B., Yan M., Wang H., Yang S., Shi D., Guo S., Liu C. (2023). Effect of Wu Zhi San supplementation in LPS-induced intestinal inflammation and barrier damage in broilers. Front. Vet. Sci..

[33] Zhu X., Shang X., Lin G., Li H., Feng X., Zhang H. (2022). Effects of Zinc Glycinate on Growth Performance, Serum Biochemical Indexes, and Intestinal Morphology of Yellow Feather Broilers. Biol. Trace Elem. Res..

[34] Liu D.Y., Lou W.J., Zhang D.Y., Sun S.Y. (2020). ROS Plays a Role in the Neonatal Rat Intestinal Barrier Damages Induced by Hyperoxia. Biomed. Res. Int..

[35] Zhang J.C., Ma L., Wan X.Y., Shan J.J., Qu Y.G., Hashimoto K.J. (2021). (R)-Ketamine attenuates LPS-induced endotoxin-derived delirium through inhibition of neuroinflammation. Psychopharmacology.

[36] Zhi Y., Li T., Li Y., Zhang T., Du M., Zhang Q., Wang X., Hu G. (2023). Protective role of Cecropin AD against LPS-induced intestinal mucosal injury in chickens. Front Immunol..

[37] Zhang L., Zhang L., Zhan X., Zeng X., Zhou L., Cao G., Chen A., Yang C. (2016). Effects of dietary supplementation of probiotic, Clostridium butyricum, on growth performance, immune response, intestinal barrier function, and digestive enzyme activity in broiler chickens challenged with Escherichia coli K88. J. Anim. Sci. Biotechnol..

[38] Wu Z., Yang K., Zhang A., Chang W., Zheng A., Chen Z., Cai H., Liu G.H. (2021). Effects of Lactobacillus acidophilus on the growth performance, immune response, and intestinal barrier function of broiler chickens challenged with Escherichia coli O157. Poult. Sci..

[39] Guo Y., Yu Y., Li H., Ding X., Li X., Jing X., Chen J., Liu G., Lin Y., Jiang C. (2021). Inulin supplementation ameliorates hyperuricemia and modulates gut microbiota in Uox-knockout mice. Eur. J. Nutr..

[40] Zhang L.H., Wang J., Piao X.S. (2022). Potential Effects of 25-Hydroxycholecalciferol on the Growth Performance, Blood Antioxidant Capacity, Intestinal Barrier Function and Microbiota in Broilers under Lipopolysaccharide Challenge. Antioxidants.

[41] Zhang B.L., Zhong Q.Z., Liu N., Song P., Zhu P., Zhang C.C., Sun Z.W. (2022). Dietary Glutamine Supplementation Alleviated Inflammation Responses and Improved Intestinal Mucosa Barrier of LPS-Challenged Broilers. Animals.

[42] Gao M., Wang J., Lv Z. (2023). Supplementing Genistein for Breeder Hens Alters the Growth Performance and Intestinal Health of Offspring. Life.

[43] Miao F., Shan C., Geng S., Ning D. (2023). Oleocanthal alleviated lipopolysaccharide-induced acute lung injury in chickens by inhibiting TLR4/NF-κB pathway activation. Poult. Sci..

[44] Dong N., Li X., Xue C., Zhang L., Wang C., Xu X., Shan A. (2020). Astragalus polysaccharides alleviates LPS-induced inflammation via the NF-κB/MAPK signaling pathway. J. Cell Physiol..

[45] Cui Y., Qu Y.Y., Yin K., Zhang X.T., Lin H.J. (2021). Selenomethionine ameliorates LPS-induced intestinal immune dysfunction in chicken jejunum. Metallomics.

[46] Kothari P., Dhaniya G., Sardar A., Sinha S., Girme A., Rai D., Chutani K., Hingorani L., Trivedi R. (2023). A glucuronated flavone TMMG spatially targets chondrocytes to alleviate cartilage degeneration through negative regulation of IL-1β. Biomed. Pharmacother..

[47] Xia C.X., Gao A.X., Zhu Y., Dong T.T., Tsim K.W. (2023). Flavonoids from Seabuckthorn (*Hippophae rhamnoides* L.) restore CUMS-induced depressive disorder and regulate the gut microbiota in mice. Food Funct..

[48] Jiang M., Lv Z., Huang Y., Cheng Z., Meng Z., Yang T., Yan Q., Lin M., Zhan K., Zhao G. (2022). Quercetin Alleviates Lipopolysaccharide-Induced Inflammatory Response in Bovine Mammary Epithelial Cells by Suppressing TLR4/NF-κB Signaling Pathway. Front. Vet. Sci..

[49] Qu Y., Li X., Xu F., Zhao S., Wu X., Wang Y., Xie J. (2021). Kaempferol Alleviates Murine Experimental Colitis by Restoring Gut Microbiota and Inhibiting the LPS-TLR4-NF-κB Axis. Front. Immunol..

[50] He C., Yu W., Yang M., Li Z., Yu J., Zhong D., Deng S., Song Z., Cheng S. (2023). Qi Fu Yin ameliorates neuroinflammation through inhibiting RAGE and TLR4/NF-κB pathway in AD model rats. Aging.

[51] Song B., Li P., Yan S., Liu Y., Gao M., Lv H., Lv Z., Guo Y.M. (2022). Effects of Dietary Astragalus Polysaccharide Supplementation on the Th17/Treg Balance and the Gut Microbiota of Broiler Chickens Challenged with Necrotic Enteritis. Front. Immunol..

[52] Adewole D., Akinyemi F. (2021). Gut Microbiota Dynamics, Growth Performance, and Gut Morphology in Broiler Chickens Fed Diets Varying in Energy Density with or without Bacitracin Methylene Disalicylate (BMD). Microorganisms.

[53] Zhao C., Hu X., Bao L., Wu K., Zhao Y., Xiang K., Li S., Wang Y., Qiu M., Feng L. (2022). Gut dysbiosis induces the development of mastitis through a reduction in host anti-inflammatory enzyme activity by endotoxemia. Microbiome.

[54] Gureev A.P., Shaforostova E.A., Vitkalova I.Y., Sadovnikova I.S., Kalinina Y.I., Cherednichenko V.R., Reznikova K.A., Valuyskikh V.V., Popov V.N. (2020). Long-term mildronate treatment increased Proteobacteria level in gut microbiome, and caused behavioral deviations and transcriptome change in liver, heart and brain of healthy mice. Toxicol. Appl. Pharmacol..

[55] Xie L., Chi X., Wang H., Dai A., Dong J., Liu S., Zhang D. (2023). Mechanism of action of buckwheat quercetin in regulating lipid metabolism and intestinal flora via Toll-like receptor 4 or nuclear factor κB pathway in rats on a high-fat diet. Nutrition.

[56] Zhou L.X., Zhang M.M., Wang Y.M., Dorfman R.G., Liu H., Yu T., Chen X., Tang D., Xu L., Yin Y. (2018). Faecalibacterium prausnitzii Produces Butyrate to Maintain Th17/Treg Balance and to Ameliorate Colorectal Colitis by Inhibiting Histone Deacetylase 1. Inflamm. Bowel Dis..

[57] Cai S., Yang Y., Kong Y., Guo Q., Xu Y., Xing P., Sun Y., Qian J., Xu R., Xie L. (2022). Gut Bacteria Erysipelatoclostridium and Its Related Metabolite Ptilosteroid a Could Predict Radiation-Induced Intestinal Injury. Front. Public Health.

[58] Sun Y., Wang F., Liu Y., Liu S., An Y., Xue H., Wang J., Xia F., Chen X., Cao Y.G. (2022). Microbiome-metabolome responses of Fuzhuan brick tea crude polysaccharides with immune-protective benefit in cyclophosphamide-induced immunosuppressive mice. Food Res. Int..

